# A Photoelectric-Stimulated MoS_2_ Transistor for Neuromorphic Engineering

**DOI:** 10.34133/2019/1618798

**Published:** 2019-11-11

**Authors:** Shuiyuan Wang, Xiang Hou, Lan Liu, Jingyu Li, Yuwei Shan, Shiwei Wu, David Wei Zhang, Peng Zhou

**Affiliations:** ^1^ASIC & System State Key Lab., School of Microelectronics, Fudan University, Shanghai 200433, China; ^2^Department of Physics, State Key Laboratory of Surface Physics, Key Laboratory of Micro and Nano Photonic Structures (Ministry of Education), and Institute for Nanoelectronic Devices and Quantum Computing, Fudan University, Shanghai 200433, China

## Abstract

The von Neumann bottleneck has spawned the rapid expansion of neuromorphic engineering and brain-like networks. Synapses serve as bridges for information transmission and connection in the biological nervous system. The direct implementation of neural networks may depend on novel materials and devices that mimic natural neuronal and synaptic behavior. By exploiting the interfacial effects between MoS_2_ and AlOx, we demonstrate that an h-BN-encapsulated MoS_2_ artificial synapse transistor can mimic the basic synaptic behaviors, including EPSC, PPF, LTP, and LTD. Efficient optoelectronic spikes enable simulation of synaptic gain, frequency, and weight plasticity. The Pavlov classical conditioning experiment was successfully simulated by electrical tuning, showing associated learning behavior. In addition, h-BN encapsulation effectively improves the environmental time stability of our devices. Our h-BN-encapsulated MoS_2_ artificial synapse provides a new paradigm for hardware implementation of neuromorphic engineering.

## 1. Introduction

The challenges of traditional computing architectures stem from storage capacity limitations and the high cost of specific data transfer speeds between memory and processors, the so-called von Neumann bottleneck [1–5]. With the advent of the artificial intelligence and big data era, this dilemma is becoming more profound. Brain-inspired neuromorphic engineering is different to the von Neumann architecture, combining memory and computation, with efficient energy utilization, and flexible adaptive and massively parallel computing capabilities [6]. It may achieve unprecedented technological breakthroughs, fundamentally overcoming the von Neumann bottleneck [7, 8]. Artificial synapses, just as those in the biological nervous system [9], play an important role in connecting various neuron blocks as the basic units of neuromorphic engineering [10]. Constructing new, stable, reliable, and efficient artificial high-performance synaptic devices is essential for neuromorphic engineering and neural network computing [11]. Many artificial synaptic devices have been reported, including oxide electric double layer [12–14], ionic liquid/gel transistors [15–20], memristors [21–29], phase-changed memory [30–34], and ferroelectric transistors [35–37]. Also, the unique internal and interfacial structures of two-dimensional (2D) materials, as well as their electrical and optical properties [38–40], make them promising candidates for complex neuromorphic engineering [41–45]. In addition, optical modulation can establish a connection between the external environment and the brain through the visual system [46–48], and combining effective optoelectronic modulation is critical for neuromorphic engineering applications, such as artificial eyes and super vision [49–51].

Here, we demonstrate an efficient photoelectrical tunable h-BN-encapsulated MoS_2_ synaptic transistor with basic synaptic functions. Furthermore, under electrical modulation, we successfully simulate the impressive Pavlov classical conditioning experiment through *V*_bg_ tuning, which realizes the acquisition, extinction, and recovery function of associated learning. Due to the h-BN encapsulation, our devices exhibit superior environmental time stability. Our h-BN-encapsulated MoS_2_ artificial synaptic transistor provides a novel paradigm for neuromorphic engineering based on 2D materials.

## 2. Results

**Figure 1:**
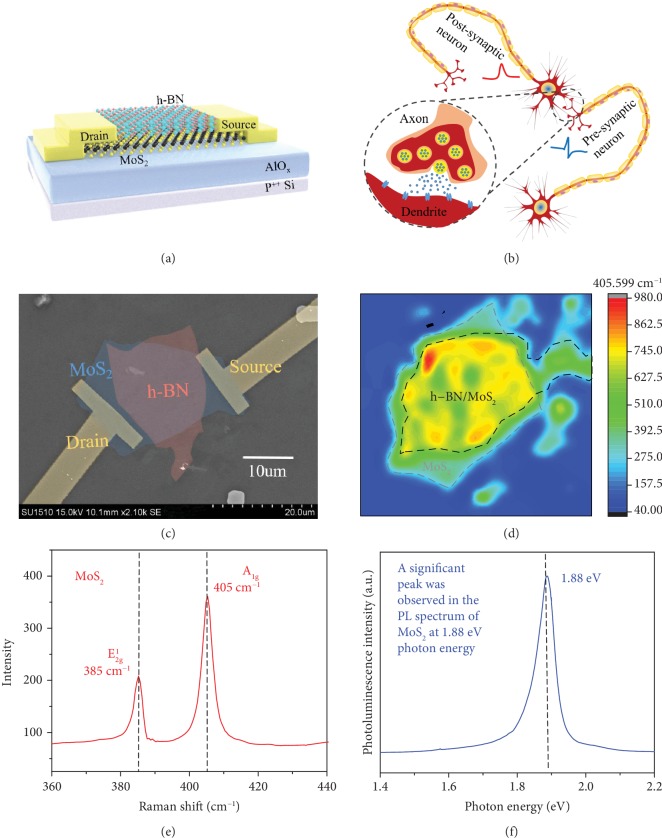
The h-BN-encapsulated MoS_2_ synaptic transistor for neuromorphic engineering. (a) Schematic of h-BN-encapsulated MoS_2_ synaptic transistor. (b) Schematic diagram of biological neurons and synapses as a bridge of neuronal connections. (c) False-color SEM image of h-BN-encapsulated MoS_2_ synaptic transistor. (d) The Raman mapping of h-BN-encapsulated MoS_2_ synaptic transistor at 405 cm^−1^, where the black and gray dashed areas represent the h-BN/MoS_2_ overlap region and channel MoS_2_, respectively. (e) Raman shift of the MoS_2_ characteristic peak is 385,405 cm^−1^. (f) A significant peak was observed in the PL spectrum of MoS_2_ at 1.88 eV photon energy, which is consistent with the band gap of multilayer MoS_2_.

**Figure 2:**
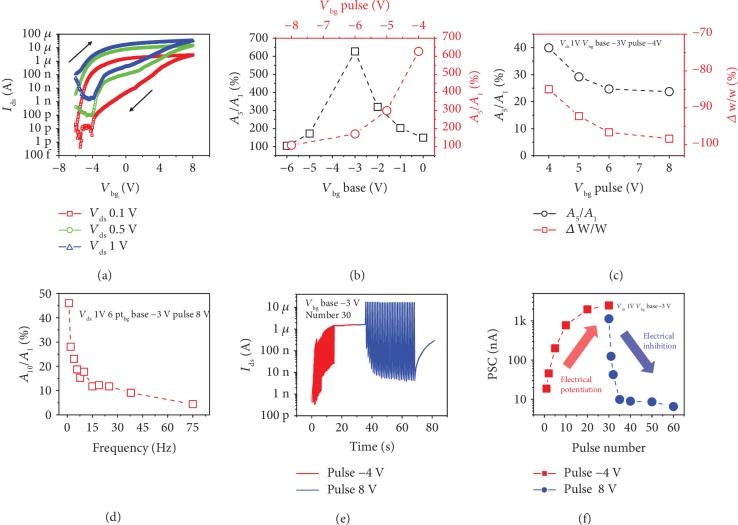
Characteristics of h-BN-encapsulated MoS_2_ synaptic transistor under electrical stimulation. (a) Transfer curve under different *V*_ds_. (b) Selecting the optimal base and pulse for the excitatory synapse by gain, when base of -3 V and pulse of -4 V, the maximum gain is obtained. (c) Selecting the optimal pulse of inhibitory synapse by gain and long-term synaptic weight change, the maximum inhibition effect and weight change are obtained when pulse is 8 V. (d) Frequency plasticity of inhibitory synapses, and the gain gradually decreases as the frequency increases. (e) Accumulation of postsynaptic current characteristics under 30 excitatory and inhibitory pulse stimulations. (f) Postsynaptic current characteristics as a function of progressive excitatory and inhibitory pulse stimulation numbers, showing long-term potentiation and inhibition effects.

First, we fabricated an h-BN-encapsulated MoS_2_ synaptic transistor on an AlO_x_/Si substrate, which simulates synaptic behavior by photoelectric stimulation, as shown in Figures 1(a) and 1(b). 2D layered h-BN and MoS_2_ were prepared by mechanical exfoliating. The surface morphology of our device was characterized by scanning electron microscopy (SEM) and atomic force microscopy (AFM), as shown in Figure 1(c) and [Supplementary-material supplementary-material-1], respectively, showing a typical channel width of 10 *μ*m, a length of 15 *μ*m, and the thickness of the MoS_2_; h-BN was approximately 1.7 and 7 nm. The Raman spectrum shows the characteristic peaks of both materials: Raman shift of the MoS_2_ characteristic peak is 385,405 cm^−1^ (Figure 1(e)) and the h-BN is 1366 cm^−1^ ([Supplementary-material supplementary-material-1]), which is consistent with previous reports. Figure 1(d) shows the Raman mapping of h-BN-encapsulated MoS_2_ synaptic transistor at 405 cm^−1^, the channel MoS_2_ exhibits intense intensity, and the h-BN/MoS_2_ overlap region is more strongly correlated with h-BN encapsulation, where the black and gray dashed areas represent the h-BN/MoS_2_ overlap region and channel MoS_2_, respectively. A significant peak was observed in the PL spectrum of MoS_2_ at 1.88 eV photon energy (Figure 1(f)), which is consistent with the band gap of multilayer MoS_2_. Then, we studied the behavioral characteristics of our h-BN-encapsulated MoS_2_ synaptic transistor under electrical modulation.  Figure 2(a) shows the *I*_ds_-*V*_bg_ curves of the h-BN-encapsulated MoS_2_ synaptic transistor with *V*_ds_ of 0.1, 0.5, and 1 V. The back gate voltage was swept from -6 to 8 V, then swept back, and a noticeable clockwise hysteresis loop was observed, which may be due to charge trapping between the MoS_2_ and AlO_x_ interfaces. The statistical distribution of the maximum value of the memory window indicates that the memory window of most devices is 2~3 V (see the statistics of 80 devices in [Supplementary-material supplementary-material-1] in Supplementary Materials). The transfer curves of the h-BN/MoS_2_/h-BN control devices show no hysteresis window, since the bottom h-BN isolates the interface effect of MoS_2_ and AlO_x_ (see Figures [Supplementary-material supplementary-material-1], the schematic diagram of the control devices, micrograph of the control device, and transfer curves of the control devices in Supplementary Materials). Owing to top encapsulated h-BN, the stability of our devices has been significantly improved (see [Supplementary-material supplementary-material-1], output curves and stability of h-BN-encapsulated MoS_2_ synaptic transistor in Supplementary Materials) [52–55]. We explored the optimal base and pulse voltages for device operation in electrical mode for excitatory and inhibitory synapses, with reference to gain (*A*_5_/*A*_1_, the amplitude of the postsynaptic current caused by spike is denoted by *A*) of five consecutive pulses and long-term synaptic weight changes (*Δ*W/W, calculated by (*I* − *I*0)/*I*0∗100%, where *I*0 and *I* represent the current states before and after the application of the pulse signal, respectively. Before applying the pulse signal, we select the average value at the 5th second as *I*0. After the pulse signal is applied, the average value of the 40th second is selected as *I*, and the pulse signals are applied at the same time. For excitatory synapses, the gain was maximized when *V*_bg_ base was -3 V and pulse was -4 V (pulse duration of 10 ms, interval of 200 ms), as shown in Figure 2(b) (no synaptic excitability of the h-BN/MoS_2_/h-BN control devices under the same *V*_bg_ base and pulse conditions, see [Supplementary-material supplementary-material-1] in Supplementary Materials). For inhibitory synapses, excitatory spike stimulation was first performed, and then fixed base, incremental *V*_bg_ pulse was applied, and gain and weight changes were reduced, that is, the depression effect gradually strengthened, and 8 V was selected as the inhibitory spike (duration of 10 ms, interval of 200 ms), as shown in Figure 2(c) (no synaptic inhibition of the h-BN/MoS_2_/h-BN control devices under the same *V*_bg_ base and pulse conditions, see [Supplementary-material supplementary-material-1] in Supplementary Materials).  Figure 2(d) shows the frequency plasticity of inhibitory synapses with fixed duration of 10 ms and number of 10, and the gain gradually decreases as the frequency increases.  Figure 2(e) depicts postsynaptic current characteristics under 30 cumulative excitatory and inhibitory spiking stimulations (duration of 10 ms, interval of 200 ms), which exhibits long-term potentiation and inhibition under electrical mode (the h-BN/MoS_2_/h-BN control devices have no LTP and LTD characteristics under the same *V*_bg_ base and pulse, see [Supplementary-material supplementary-material-1] in Supplementary Materials). Furthermore, Figure 2(f) shows extracted PSC from excitatory and inhibitory spikes, where electrical potentiation and inhibition are clearly observed. The number-dependent facilitation and depression under electrical stimulation are shown in [Supplementary-material supplementary-material-1] in Supplementary Materials. The electrical potentiation and inhibition effects under electrical stimulation are attributed to the charges trapping and detrapping at the MoS_2_-AlO_x_ interfaces. The statistical distribution of the maximum value of the excitatory index indicates that the excitatory index of most devices can reach 500-700% (see the statistics of 80 devices in [Supplementary-material supplementary-material-1] in Supplementary Materials). Under forward bias (*V*_bg_ pulse of 8 V), the oxygen vacancy trapping states in AlO_x_ move toward the channel, trapping the electrons in MoS_2_, causing channel current to decrease, corresponding to synaptic inhibition. While under reverse bias (*V*_bg_ pulse of -4 V), oxygen ions in AlO_x_ move toward MoS_2_, and the oxygen vacancy trapping states release trapped electrons, resulting in increased channel current, which corresponds to synaptic potentiation (see [Supplementary-material supplementary-material-1], physical mechanism under electrical stimulation in Supplementary Materials).

**Figure 3:**
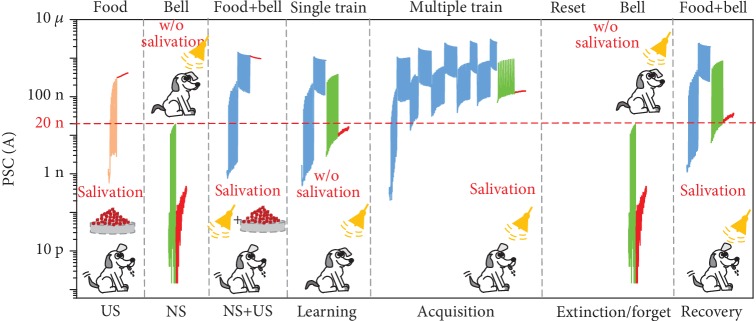
Pavlov's dog classical conditioning experiment implemented by h-BN-encapsulated MoS_2_ synaptic transistor. Pavlov's dog classical conditioning experiments can be simulated on the proposed h-BN-encapsulated MoS_2_ synaptic transistor by efficient electrical modulation. *V*_bg_ (base, pulse) of (-5, -4 V) applied to the presynaptic gate is considered to be “bell” (NS), and *V*_bg_ (base, pulse) of (-3, -4 V) is considered “food” (US). The postsynaptic source drain channel current acts as synaptic weight, and the synaptic weight of 20 nA is defined as the threshold for the “salivation” response.

The realization of the association learning is of great significance for neuromorphic engineering. Pavlov's dog classical conditioning experiment is a typical associative learning experiment in physiology [56–58]. In Pavlov's dog experiment, food is called unconditional stimulation (US), while the bell and salivation are called neutral stimulation (NS) and unconditional response (UR), respectively. Food can cause salivation, while bell ringing alone does not cause salivation. Combining the bell with food, that is, after the bell rings, the dog is fed with food, also causes salivation [57, 59]. Pavlov's dog classical conditioning experiment can be simulated on the proposed h-BN-encapsulated MoS_2_ synaptic transistor by efficient electrical modulation, as shown in Figure 3. *V*_bg_ (base, pulse) of (-5, -4 V) applied to the presynaptic gate is considered to be “bell” (NS), and *V*_bg_ (base, pulse) of (-3, -4 V) is considered “food” (US). The postsynaptic source drain channel current acts as synaptic weight, and the synaptic weight of 20 nA is defined as the threshold for the “salivation” response (UR). After a single training, only the “bell” ringing does not cause salivation, but after repeated training, the “bell” ringing can also cause “salivation,” which shows the same effect as feeding “food.” At this point, an association is established between “bell” and “food,” and the corresponding NS “bell” is converted to conditional stimulation (CS), causing a conditional response (CR) that triggers “salivation” similar to US, which is called acquisition. After a long time or reset operation, “salivation” no longer occurs when there is only CS, which means that the association between CS and US is extinct/forgotten. However, after training again, “salivation” occurs again when the “bell” rings only, that is, the association is recovered. In addition, we found that due to the existence of acquisition, the current of single training after recovery is significantly higher than the previous single training, which has exceeded the threshold and “salivation” occurs.

**Figure 4:**
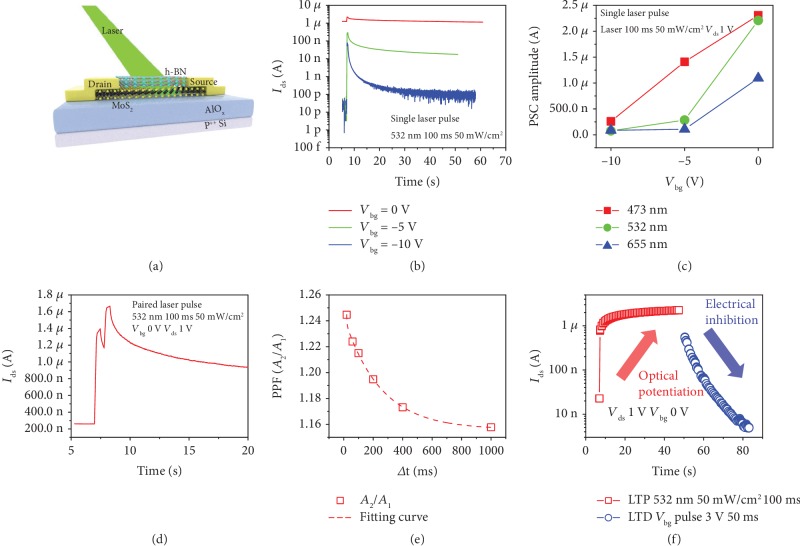
Basic synaptic characteristics of h-BN-encapsulated MoS_2_ transistor under optical stimulation. (a) Schematic diagram of h-BN-encapsulated MoS_2_ synaptic transistor under optical stimulus. (b) Single-laser pulse characteristics under different *V*_bg_ (0, -5, -10 V). (c) Variation of postsynaptic current amplitude under different *V*_bg_ (0, -5, -10 V) and single-laser pulses of different wavelengths (473, 532, 655 nm). (d) Typical paired laser pulse facilitation characteristics. (e) PPF characteristics as a function of paired laser pulse intervals. (f) Postsynaptic current characteristics are a function of excitatory laser pulses and inhibitory electrical pulse stimulation, which also shows long-term potentiation and depression.

In addition to electrical modulation, optical spikes also enable efficient regulation of our h-BN-encapsulated MoS_2_ synaptic transistor, which uses laser pulses as the photogate to adjust the channel conductance (synaptic weight), as shown in Figure 4(a). Figure 4(b) shows the single-laser pulse characteristics (532 nm, duration of 100 ms, power of 50 mW/cm^2^) of the synaptic transistor at *V*_bg_ of 0, -5, and -10 V, which significantly affect the reference current (the single-laser pulse characteristics of our synaptic transistor at 473,655 nm and the single-laser pulse current versus time at three wavelengths with *V*_ds_ of 1 V are detailed in [Supplementary-material supplementary-material-1] in Supplementary Materials). Besides, variation of postsynaptic current amplitude under different *V*_bg_(0, -5, -10 V) and single-laser pulses with different wavelengths (473, 532, 655 nm) is shown in Figure 4(c). We found that the PSC amplitude increases significantly with *V*_bg_, where different wavelengths have little effect on the PSC amplitude (both of *μ*A), which may be due to the excitation of the h-BN-encapsulated MoS_2_ synaptic transistor at each wavelength, resulting in photocarrier accumulation in the channel. Specifically, photogenerated carriers (electron-hole pairs) are generated and separated in the top h-BN under laser duration, in which photogenerated electrons are transferred to MoS_2_, resulting in an increase in channel current. With the cumulative number of laser pulses, the electrons in MoS_2_ increase continuously, and the channel current appears to be nonvolatile, corresponding to the LTP behavior of neural synapses (see the physical mechanism under optical stimulation in [Supplementary-material supplementary-material-1] in Supplementary Materials). Moreover, paired pulse facilitation (PPF) is a dynamic increase in neurotransmitter release that is thought to be critical in biosynaptic function simulations [60], where presynaptic-induced EPSC amplitude decreases with increasing two consecutive pulse intervals (Δ*t*). Figure 4(d) describes the postsynaptic current response when a pair of consecutive laser pulses (532 nm, duration of 100 ms, 50 mW/cm^2^ of power, *V*_bg_ and *V*_ds_ are 0 and 1 V) is applied. For small Δ*t*, the postsynaptic current is further enhanced, resulting in *A*_2_ > *A*_1_, corresponding to typical synaptic PPF characteristics. Figure 4(e) exhibits the PPF index *A*_2_/*A*_1_ as a function of the interval time (Δ*t*), where the red dashed line represents the fitting curve of the double exponential decay function (Equation (1)) [61]. *C*_1_ and *C*_2_ are the initial magnitudes of the fast and slow phases, and *t*_1_ and *t*_2_ are the characteristic relaxation times of the phases. For our h-BN-encapsulated MoS_2_ synaptic transistor, *t*_1_ and *t*_2_ are about 3.0 and 247.4 ms, respectively, which is faster than in most previous work and is consistent with the relaxation time in biological synapses [16, 20, 62–65]. Moreover, we demonstrate long-term synaptic potentiation and inhibition effects under photoelectric modulation with *V*_bg_and *V*_ds_ of 0 and 1 V, i.e., implementing optical potentiation (532 nm, laser duration 100 ms, power 50 mW/cm^2^) and electrical inhibition (*V*_bg_ pulse 3 V, duration 50 ms) behaviors in sequence, as shown in Figure 4(f). The optimal *V*_bg_ pulses for inhibition under optical stimulation are explored in [Supplementary-material supplementary-material-1] in Supplementary Materials, and 50 laser-stimulated LTP, followed by 50 electrical stimulation LTD characteristics, are shown in [Supplementary-material supplementary-material-1] of Supplementary Materials, respectively.

(1) $$\begin{array}{c} \text{PPF}\left(\frac{A_{2}}{A_{1}}\right)=C_{1}*\exp\left(\frac{-\Delta t}{t_{1}}\right)+C_{2}*\exp\left(\frac{-\Delta t}{t_{2}}\right)+C_{0}. \end{array}$$

**Figure 5:**
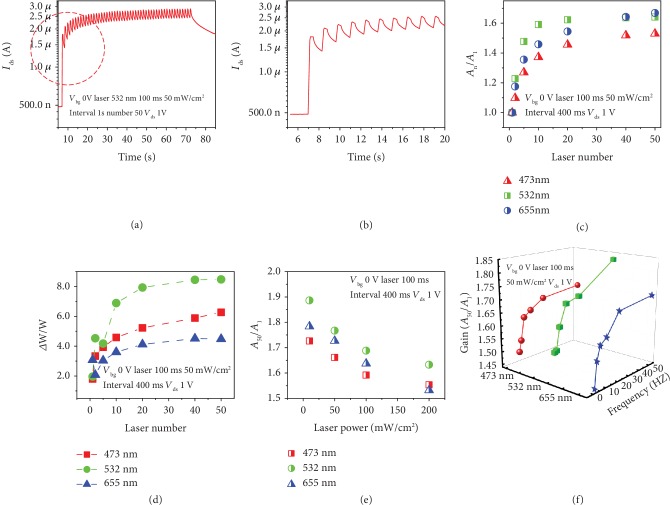
Optical neural plasticity of h-BN-encapsulated MoS_2_ synaptic transistor. (a) Typical long-term potentiation of h-BN-encapsulated MoS_2_ synaptic transistor under optical stimulation. (b) Magnification of the dotted circle area in (a). (c) Gain variation of different wavelengths (473, 532, 655 nm) and pulse numbers under laser stimulation. (d) Long-term synaptic weight changes at different wavelengths (473, 532, 655 nm) and pulse numbers under laser stimulation. (e) Gain variation of different wavelengths and laser powers under optical modulation. (f) Gain as a function of laser wavelength and frequency in optical modulation mode.

Subsequently, we demonstrate the optical neural plasticity of the h-BN-encapsulated MoS_2_ synaptic transistor.  Figure 5(a) exhibits a typical synaptic LTP of our h-BN-encapsulated MoS_2_ synaptic transistor under optical stimulation (532 nm, laser duration and intervals are 100 ms and 1 s, power of 50 mW/cm^2^, laser number of 50, *V*_bg_ and *V*_ds_ are 0 and 1 V), and Figure 5(b) is an enlargement of the dotted circle region in Figure 5(a). Gain (*A*_*n*_/*A*_1_) variation of different wavelengths (473, 532, 655 nm) and pulse numbers under laser stimulation (532 nm, laser duration and interval are 100 and 400 ms, power of 50 mW/cm^2^, *V*_bg_ and *V*_ds_ are 0 and 1 V) are demonstrated in Figure 5(c), which accumulates the laser pulse numbers, and the gains under three wavelength stimuli gradually increase and tend to saturate. Besides, we demonstrate that with the increase of the laser spiking number, the long-term synaptic weight changes at different wavelengths also gradually increase, indicating the synaptic connections are strengthened, as shown in Figure 5(d). And we found that the *Δ*W/W induced by the 532 nm laser spike is the largest, that is, the strongest synaptic connection strength, and the weakest was at 655 nm, which may be attributed to the larger the wavelength, the smaller the energy under the same conditions, and the fewer photogenerated carriers are generated, resulting in the weakest connection strength. However, 532 nm may more easily excite our h-BN-encapsulated MoS_2_ synaptic transistor than 473 nm, by concentrating more photogenerated carriers.  Figure 5(e) shows the gain (*A*_50_/*A*_1_) variation of different wavelengths and laser powers under optical modulation (laser duration and interval are 100 and 400 ms, *V*_bg_ and *V*_ds_ are 0 and 1 V). Abnormally, as the laser power increases, the synaptic gain decreases, which may be attributed to the incremental power intensity causing slight damage to the channel material and degradation of performance. Finally, we also demonstrate the synaptic gain as a function of wavelength and laser frequency (1-50 Hz, duration of 100 ms, power of 50 mW/cm^2^, *V*_bg_ and *V*_ds_ are 0 and 1 V), which increases with frequency and has a maximum at 50 Hz, as shown in Figure 5(f). PSC, PPF, gain, and *Δ*W/W tuning all demonstrate the flexibility and diversity of synaptic plasticity in our h-BN-encapsulated MoS_2_ synaptic transistor. Besides, comparing the performance of h-BN-encapsulated MoS_2_ artificial synapse with other 2D-based artificial synaptic devices, including organic and inorganic materials (such as PEDOT:PSS, CsPbBr3, Pentacene, EMIM-TFSI, PVA, MoS_2_, WSe_2_, graphene, h-BN, and BP) demonstrates the superiority of our devices (see [Supplementary-material supplementary-material-1] in Supplementary Materials). The acceptable switching power consumption is estimated to be 80 pJ per spike, which is two orders of magnitude lower than the traditional CMOS [66] and even down to femtojoule when the *V*_ds_ is 0.1 V, close to the human brain [16]. The h-BN-encapsulated MoS_2_ artificial synaptic transistor provides a novel paradigm for neuromorphic engineering based on 2D materials.

## 3. Discussion

In conclusion, our breakthrough, efficient, photoelectrical tunable, diverse h-BN-encapsulated MoS_2_ synaptic transistor demonstrates basic synaptic functions including EPSC, PPF, LTP, LTD, synaptic gain, frequency, and weight plasticity. In addition, under electrical modulation, we successfully simulated the Pavlov classical conditioning experiment and realized the associated learning function. It is worth mentioning that due to the h-BN encapsulation, our devices have superior environmental stability. Our synaptic transistor provides an unparalleled perspective on novel 2D material-based neuromorphic engineering and brain-like computing.

## 4. Materials and Methods

### 4.1. Preparation of the h-BN-Encapsulated MoS_2_ Synaptic Transistor

We fabricated the h-BN-encapsulated MoS_2_ synaptic transistor on an AlO_x_/Si substrate, which simulates synaptic behavior by photoelectric stimulation, as shown in Figures 1(a) and 1(b). Firstly, two-dimensional layered h-BN and MoS_2_ were prepared by mechanical exfoliation, and their thicknesses were determined by an atomic force microscope (AFM) to be about 7 and 1.7 nm, as shown in [Supplementary-material supplementary-material-1]. The h-BN was placed on top of the MoS_2_ by wet transfer using polyvinyl alcohol (PVA) as a sacrificial layer to construct an h-BN/MoS_2_ heterojunction and depositing the 30 nm source-drain electrodes by electron beam evaporation (EBE) to form the synaptic transistor. The detailed manufacturing process of the h-BN-encapsulated MoS_2_ synaptic transistor is shown in [Supplementary-material supplementary-material-1].

### 4.2. Device Characterization and Measurement

The surface morphology of our device was characterized by scanning electron microscopy (SEM) and atomic force microscopy (AFM), as shown in Figure 1(c) and [Supplementary-material supplementary-material-1], respectively, showing a typical channel width of 10 *μ*m, a length of 15 *μ*m, and the thickness of the MoS_2_; h-BN was approximately 1.7 and 7 nm. Besides, the 2D layered material was characterized by Raman and PL spectroscopy, as shown in Figures 1(e) and 1(f) and [Supplementary-material supplementary-material-1]. MoS_2_ shows two strong peaks near 385 and 405 cm^−1^, corresponding to the in-plane (*E*_2g_) and vertical (*A*_1g_) vibration models. The Raman spectrum of h-BN reveals a peak near 1366 cm^−1^ and also corresponds to the in-plane (*E*_2g_) vibration model. The Raman mapping of h-BN-encapsulated MoS_2_ synaptic transistor at 405 cm^−1^ is shown in Figure 1(d), the channel MoS_2_ exhibits intense intensity, and the h-BN/MoS_2_ overlap region is more strongly correlated with h-BN encapsulation, where the black and gray dashed areas represent the h-BN/MoS_2_ overlap region and channel MoS_2_, respectively. A distinct peak was observed in the PL spectrum of MoS_2_ at 1.88 eV photon energy, which is consistent with the band gap of multilayer MoS_2_. All electrical measurements of our device were performed on the cascade probe station and Keithley 4200A semiconductor analyzer, and optical measurements were performed on the TTL/analog-modulated multiwavelength (655, 532, 473 nm) laser system.

## Supplementary Materials

Supplementary Materials Figure S1: fabrication process scheme of h-BN-encapsulated MoS_2_ synaptic transistors. It is worth mentioning that it is necessary to grow a 1 nm Al seed layer and naturally oxidize for 24 hours before depositing 30 nm Al_2_O_3_ as a back gate dielectric by ALD [1–3]. Figure S2: AFM image of synaptic transistor and Raman shift characterization of h-BN. (a) The AFM image of the MoS_2_ synapse transistor in h-BN package, in which the MoS_2_ and h-BN heights are 1.7 and 7 nm, respectively. (b) Raman shift of the h-BN characteristic peak is 1366 cm^−1^. Figure S3: output characteristics and stability of h-BN-encapsulated MoS_2_ synaptic transistors. (a) *I*_ds_-*V*_ds_ curves and *V*_bg_ from -5 to 5 V in steps of 2.5 V. (b) h-BN-encapsulated MoS_2_ synaptic transistors with good time and operating stability [4–7]. Figure S4: number-dependent facilitation and depression under electrical stimulation. (a) Excitatory PSC and gain under different electrical pulse numbers. (b) PSC and inhibitory ratio under different electrical pulse numbers. Figure S5: physical mechanism under electrical stimulation. (a) Under forward bias, the oxygen vacancy trapping states in AlOx move toward the channel, trapping the electrons in MoS_2_, causing channel current to decrease. (b) Under reverse bias, oxygen ions in AlOx move toward MoS_2_, and the oxygen vacancy trapping states release trapped electrons, resulting in increased channel current [8–10]. Figure S6: single pulse characteristics of h-BN-encapsulated MoS_2_ synaptic transistors under different *V*_bg_ and wavelength lasers. (a) Characteristics of different *V*_bg_ (0, -5, -10 V) under a single 473 nm laser pulse. (b) Characteristics of different *V*_bg_ under a single 655 nm laser pulse. (c) Characteristics of single-laser pulses of different wavelengths under *V*_bg_ 0 V. Figure S7: optimal *V*_bg_ pulse for inhibition under optical stimulation. (a) 2 V of *V*_bg_ pulse. (b) 3 V of *V*_bg_ pulse. (c) 4 V of *V*_bg_ pulse. (d) 6 V of *V*_bg_ pulse. Figure S8: LTP and LTD behaviors under optical/electrical stimulation. (a) LTP behavior under 50 laser pulses. (b) Subsequent LTD behavior for 50 electrical pulses. Figure S9: physical mechanism under optical stimulation. (a) Photogenerated carriers (electron-hole pairs) are generated and separated in the top h-BN under laser duration, in which photogenerated electrons are transferred to MoS_2_, resulting in an increase in channel current. (b) With the cumulative number of laser pulses, the electrons in MoS_2_ increase continuously, and the channel current appears to be nonvolatile, that is, LTP behavior [11]. Figure S10: characteristics of the control devices: h-BN/MoS2/h-BN structure. (a) Schematic diagram of the control devices. (b) Micrograph of a typical control device. (c) Transfer curves of the control devices. (d) No synaptic excitability of the control devices under the same *V*_bg_ base and pulse conditions. (e) No synaptic inhibition of the control devices under the same *V*_bg_ base and pulse conditions. (f) Predictably, the control devices have no LTP and LTD characteristics under the same *V*_bg_ base and pulse. Figure S11: memory window and excitatory index statistics for 80 h-BN-encapsulated MoS_2_ synaptic transistor. (a) The statistical distribution of the maximum value of the memory window (mean = 2.36, SD = 0.78), showing that the memory window is 2~3 V in most devices. (b) The excitability index of most devices can reach 500~700% (mean = 540.69, SD = 142.06). Table S1: comparison of 2D-based synaptic device performance, including device geometry, operating modes, electrical/optical tuning, excitatory index, long-term weight change, and power consumption.
