# Supplementary material for: A Photoelectric-Stimulated MoS2 Transistor for Neuromorphic Engineering
Source: Research (Wash D C). 2019 Nov 11;2019:1618798. doi: 10.34133/2019/1618798 (PMC6946262; doi:10.34133/2019/1618798)
Supplement: Supplementary Materials — Figure S1: fabrication process scheme of h-BN-encapsulated MoS2 synaptic transistors. It is worth mentioning that it is necessary to grow a 1 nm Al seed layer and naturally oxidize for 24 hours before depositing 30 nm Al2O3 as a back gate dielectric by ALD [1–3]. Figure S2: AFM image of synaptic transistor and Raman shift characterization of h-BN. (a) The AFM image of the MoS2 synapse transistor in h-BN package, in which the MoS2 and h-BN heights are 1.7 and 7 nm, respectively. (b) Raman shift of the h-BN characteristic peak is 1366 cm−1. Figure S3: output characteristics and stability of h-BN-encapsulated MoS2 synaptic transistors. (a) Ids-Vds curves and Vbg from -5 to 5 V in steps of 2.5 V. (b) h-BN-encapsulated MoS2 synaptic transistors with good time and operating stability [4–7]. Figure S4: number-dependent facilitation and depression under electrical stimulation. (a) Excitatory PSC and gain under different electrical pulse numbers. (b) PSC and inhibitory ratio under different electrical pulse numbers. Figure S5: physical mechanism under electrical stimulation. (a) Under forward bias, the oxygen vacancy trapping states in AlOx move toward the channel, trapping the electrons in MoS2, causing channel current to decrease. (b) Under reverse bias, oxygen ions in AlOx move toward MoS2, and the oxygen vacancy trapping states release trapped electrons, resulting in increased channel current [8–10]. Figure S6: single pulse characteristics of h-BN-encapsulated MoS2 synaptic transistors under different Vbg and wavelength lasers. (a) Characteristics of different Vbg (0, -5, -10 V) under a single 473 nm laser pulse. (b) Characteristics of different Vbg under a single 655 nm laser pulse. (c) Characteristics of single-laser pulses of different wavelengths under Vbg 0 V. Figure S7: optimal Vbg pulse for inhibition under optical stimulation. (a) 2 V of Vbg pulse. (b) 3 V of Vbg pulse. (c) 4 V of Vbg pulse. (d) 6 V of Vbg pulse. Figure S8: LTP and LTD behaviors under optical/ele [file 1618798.f1.docx]

Supplementary Materials

**A Photoelectric Stimulated MoS_2_ Transistor for Neuromorphic Engineering**

Shuiyuan Wang^1^, Xiang Hou^1^, Lan Liu^1^, JingYu Li^1^, Yuwei Shan^2^, Shiwei Wu^2^, David Wei Zhang^1^, Peng Zhou^1,*^

*^1^ASIC & System State Key Lab., School of Microelectronics, Fudan University, Shanghai 200433, China.*

*^2^Department of Physics, State Key Laboratory of Surface Physics, Key Laboratory of Micro and Nano Photonic Structures (Ministry of Education), and Institute for Nanoelectronic Devices and Quantum Computing, Fudan University, Shanghai 200433, China.*

** Correspondence should be addressed to Peng Zhou:* [*pengzhou@fudan.edu.cn*](mailto:pengzhou@fudan.edu.cn)


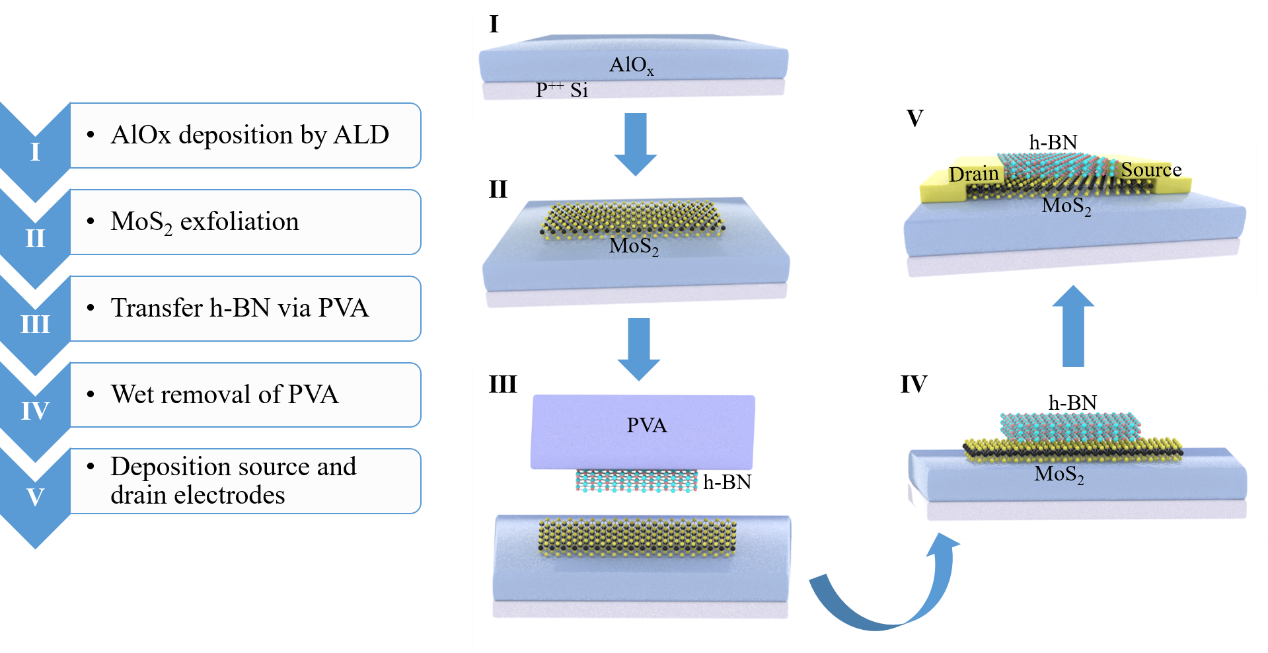


**Figure S1**. Fabrication process scheme of h-BN encapsulated MoS_2_ synaptic transistors. It is worth mentioning that it is necessary to grow a 1 nm Al seed layer and naturally oxidize for 24 hours before depositing 30 nm Al_2_O_3_ as a back gate dielectric by ALD [1-3].


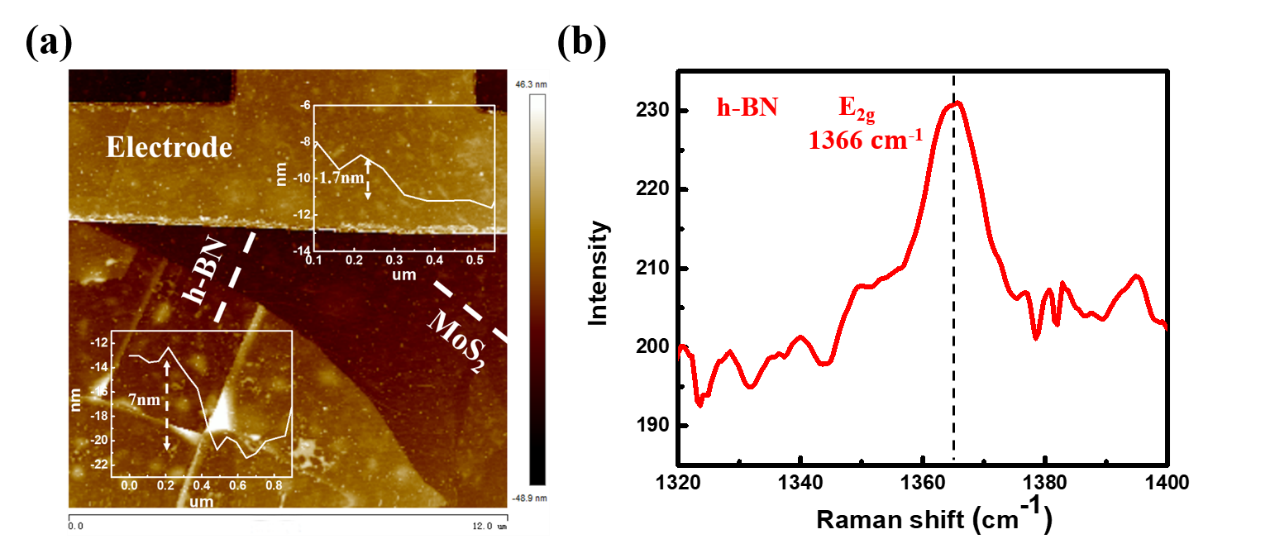


**Figure S2**. AFM image of synaptic transistor and Raman shift characterization of h-BN. (a) The AFM image of the MoS_2_ synapse transistor in h-BN package, in which the MoS_2_, h-BN heights are 1.7, 7 nm, respectively. (b) Raman shift of the h-BN characteristic peak is 1366 cm^-1^.


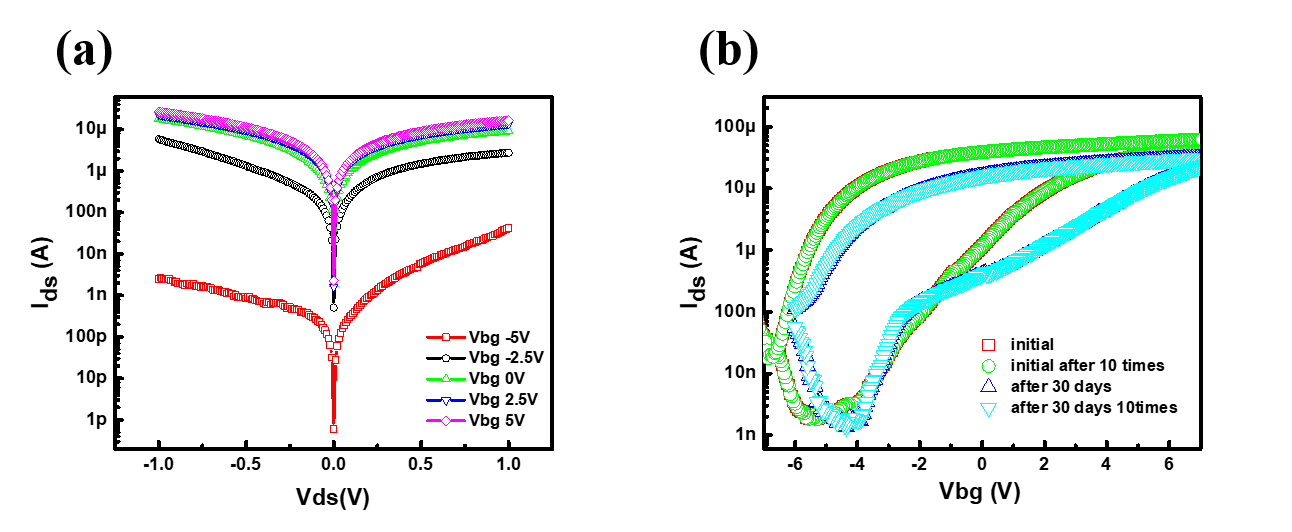


**Figure S3**. Output characteristics and stability of h-BN encapsulated MoS_2_ synaptic transistors. (a) I_ds_-V_ds_ curves, V_bg_ from -5 to 5 V in steps of 2.5 V. (b) h-BN encapsulated MoS_2_ synaptic transistors with good time and operating stability[4-7].


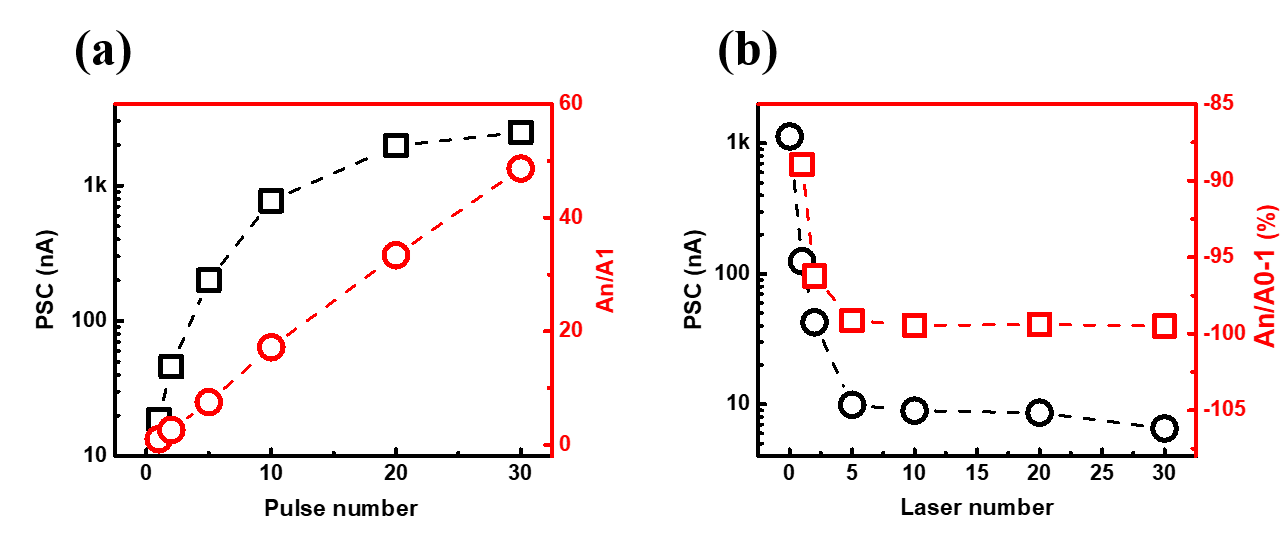


**Figure S4**. Number dependent facilitation and depression under electrical stimulation. (a) Excitatory PSC and gain under different electrical pulse numbers. (b) PSC and inhibitory ratio under different electrical pulse numbers.


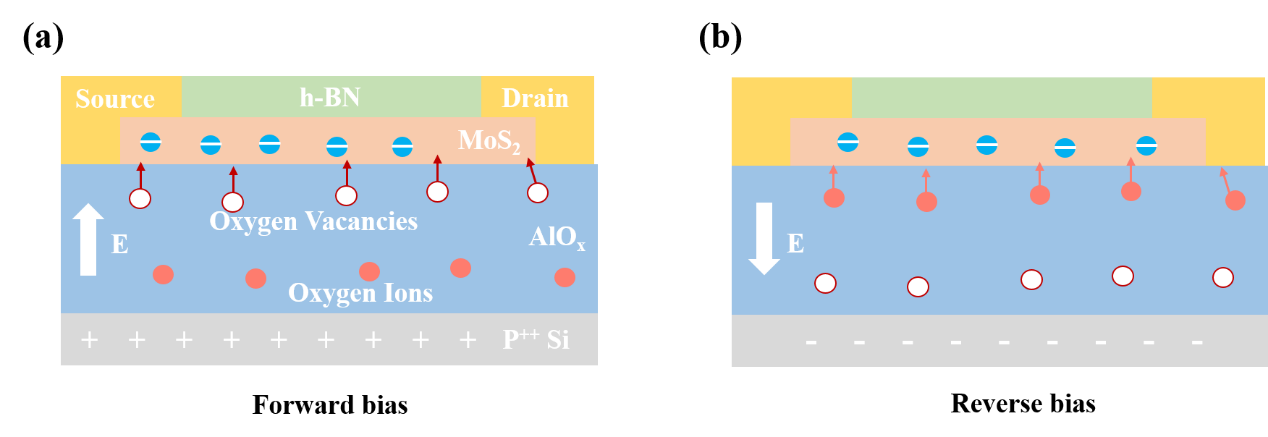


**Figure S5**. Physical mechanism under electrical stimulation. (a) Under forward bias, the oxygen vacancy trapping states in AlO_x_ move toward the channel, trapping the electrons in MoS_2_, causing channel current to decrease. (b) Under reverse bias, oxygen ions in AlO_x_ move toward MoS_2_, and the oxygen vacancy trapping states release trapped electrons, resulting in increased channel current [8-10].


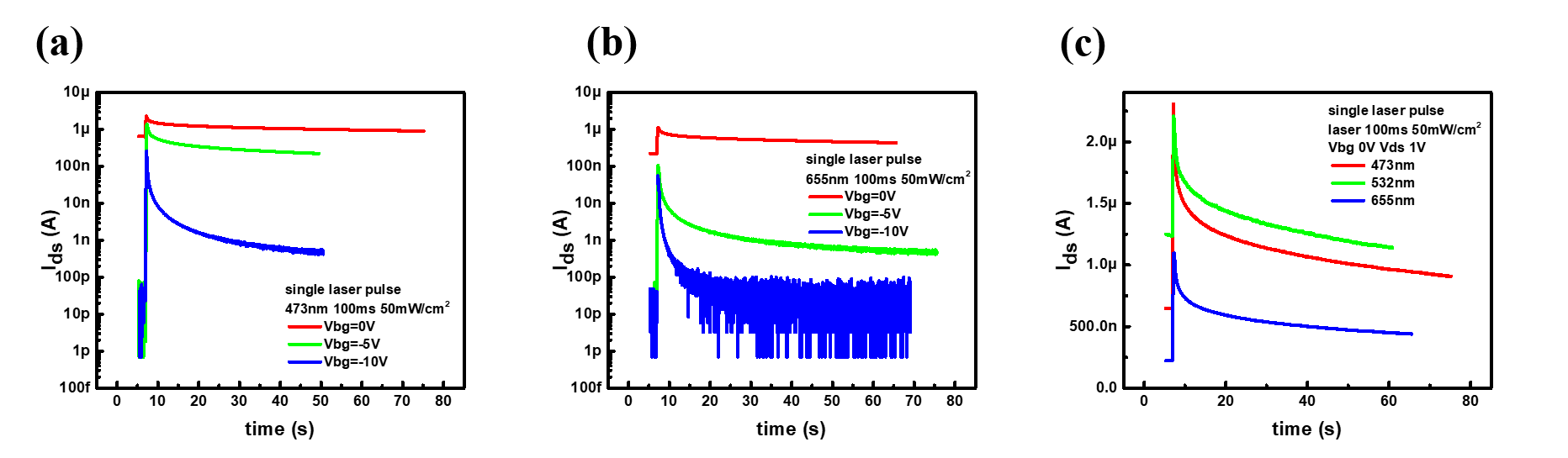


**Figure S6**. Single pulse characteristics of h-BN encapsulated MoS_2_ synaptic transistors under different V_bg_ and wavelength lasers. (a) Characteristics of different V_bg_ (0, -5, -10 V) under a single 473 nm laser pulse. (b) Characteristics of different V_bg_ under a single 655 nm laser pulse. (c) Characteristics of single laser pulses of different wavelengths under V_bg_ 0 V.


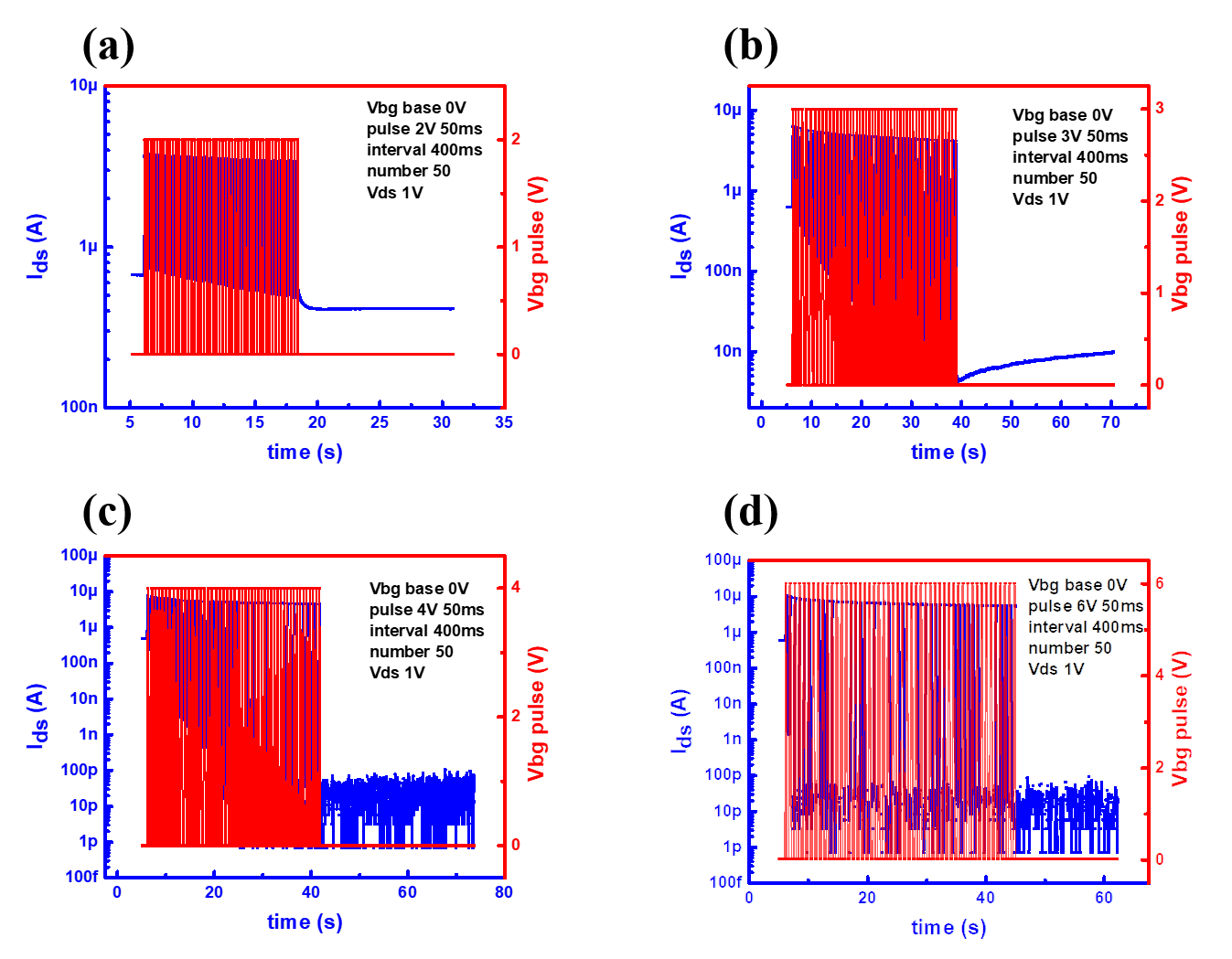


**Figure S7**. Optimal V_bg_ pulse for inhibition under optical stimulation. (a) 2 V of V_bg_ pulse. (b) 3 V of V_bg_ pulse. (c) 4 V of V_bg_ pulse. (d) 6 V of V_bg_ pulse.


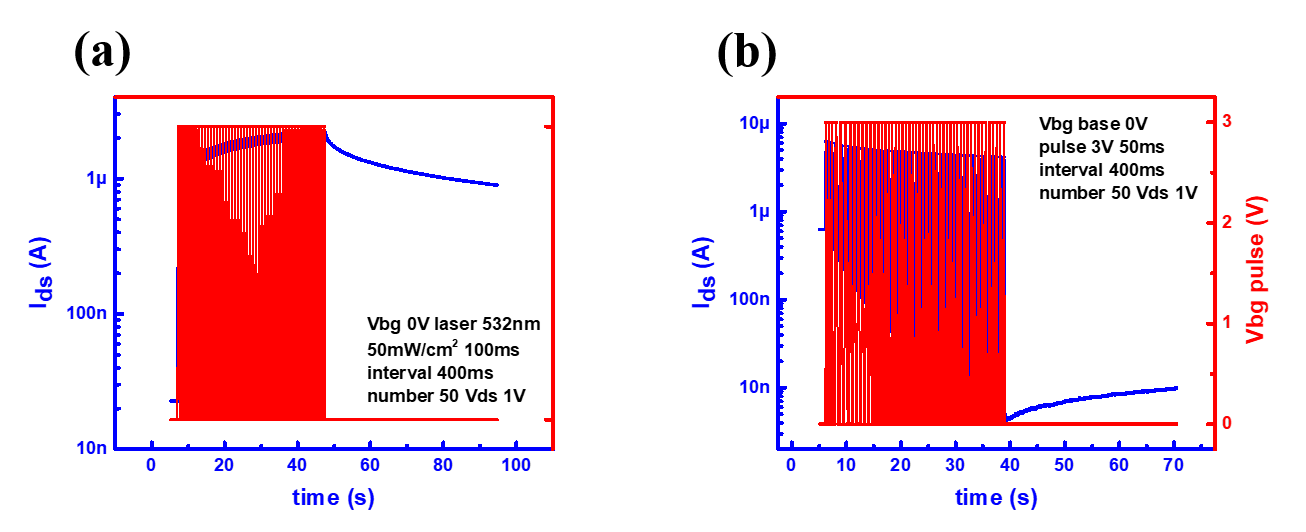


**Figure S8**. LTP and LTD behaviors under optical/electrical stimulation. (a) LTP behavior under 50 laser pulses. (b) Subsequent LTD behavior for 50 electrical pulses.


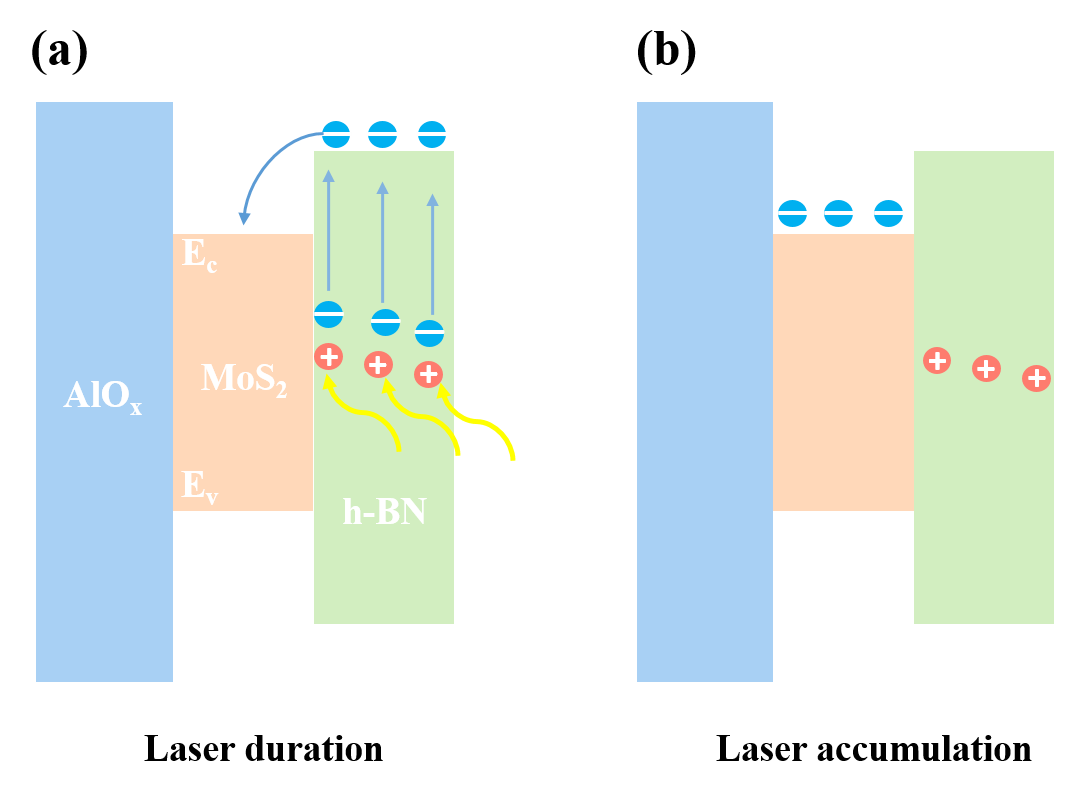


**Figure S9**. Physical mechanism under optical stimulation. (a) Photo-generated carriers (electron-hole pairs) are generated and separated in the top h-BN under laser duration, in which photo-generated electrons are transferred to MoS_2_, resulting in an increase in channel current. (b) With the cumulative number of laser pulses, the electrons in MoS_2_ increase continuously, and the channel current appears to be non-volatile, that is, LTP behavior [11].


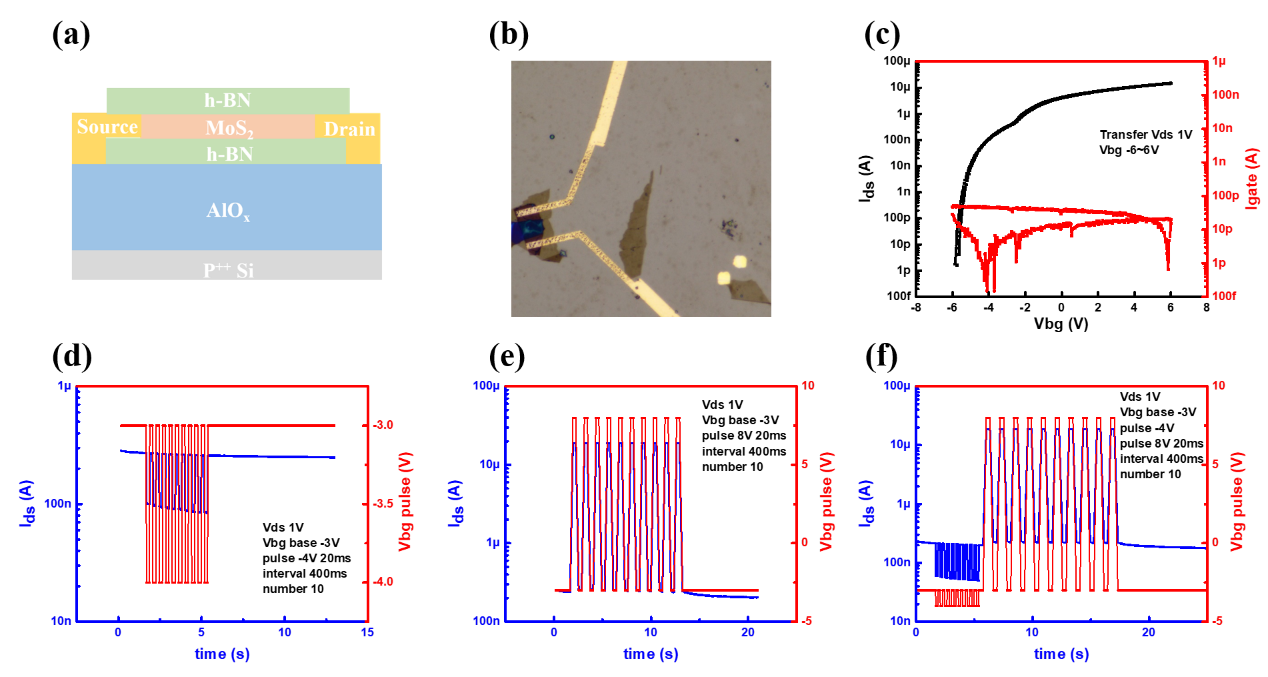


**Figure S10**. Characteristics of the control devices: h-BN/MoS_2_/h-BN structure. (a) Schematic diagram of the control devices. (b) Micrograph of a typical control device. (c) Transfer curves of the control devices. (d) No synaptic excitability of the control devices under the same V_bg_ base and pulse conditions. (e) No synaptic inhibition of the control devices under the same V_bg_ base and pulse conditions. (f) Predictably, the control devices have no LTP and LTD characteristics under the same V_bg_ base and pulse.


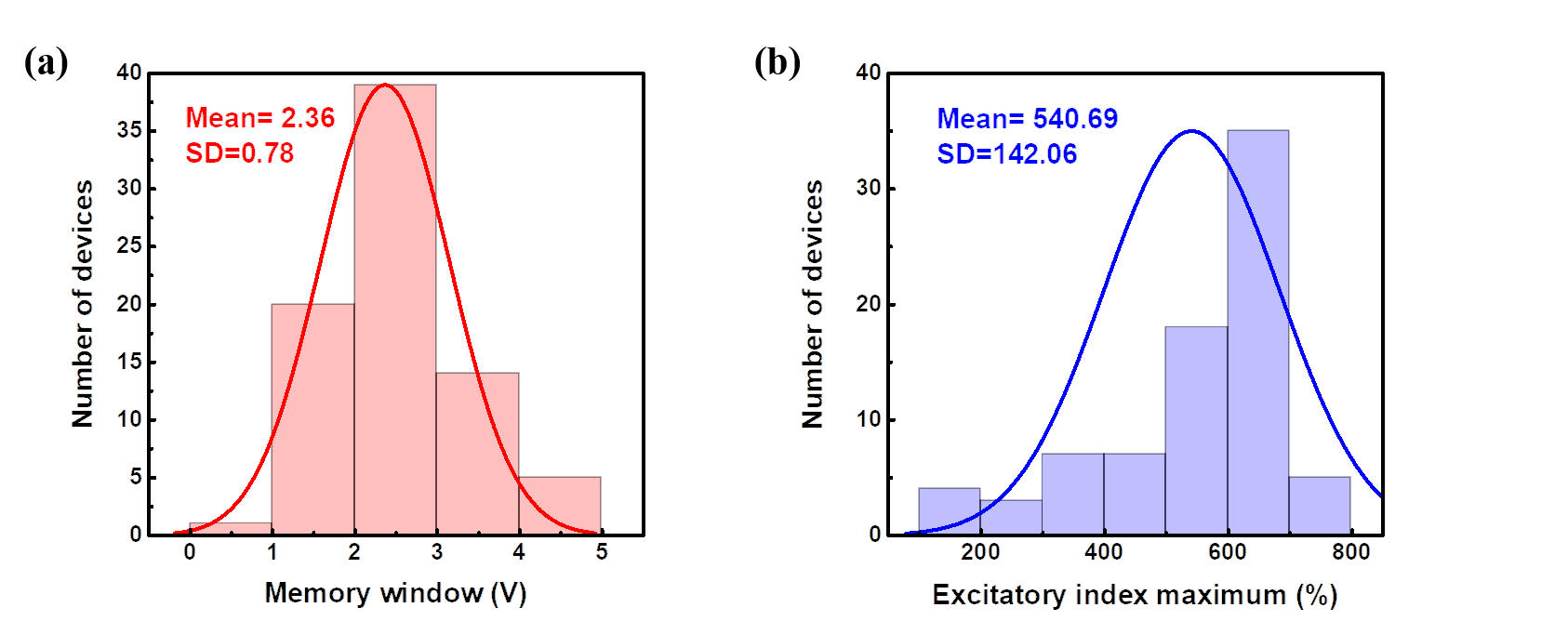


**Figure S11.** Memory window and excitatory index statistics for 80 h-BN encapsulated MoS_2_ synaptic transistor. (a) The statistical distribution of the maximum value of the memory window (Mean=2.36, SD=0.78), showing that the memory window is 2~3V in most devices (b) The excitability index of most devices can reach 500~700% (Mean=540.69, SD=142.06).

**Table S1.** Comparison of 2D-based synaptic device performance, including device geometry, operating modes, electrical/optical tuning, excitatory index, long-term weight change and power consumption.

| **Device geometry** | **Operating Modes** | **Electrical**  **tuning** | **Optical**  **tuning** | **Excitatory index (%)** | **Long-term weight change (%)** | **Power consumption per spike(pJ)** |
| --- | --- | --- | --- | --- | --- | --- |
| **h-BN/MoS_2_** | **Transistor** | √ | √ | **600** | **800** | **80** |
| PEDOT:PSS/ PEDOT:PSS/PEI[12] | Transistor | √ | × | 375 | N. A. | 10 |
| MoS_2_/PVA[13] | Transistor | √ | × | 367 | N. A. | 23.6 |
| SWNT/Gr[14] | Transistor | √ | √ | 350 | 450 | 250000 |
| W/MoS_2_/p-Si[15] | Memristor | √ | √ | 233 | 300 | N. A. |
| WSe_2_/PEO:LiClO_4_[16] | Transistor | √ | × | 220 | 500 | 1 |
| CsPbBr_3_/PMMA/ pentacene[17] | Transistor | √ | √ | 218 | N. A. | 1400 |
| MoS_2_/DEMETFSI[18] | Transistor | √ | √ | 206 | 800 | 4.8 |
| MoS_2_(Joule heating)[19] | Transistor | √ | × | 180 | N. A. | 0.01 |
| α-MoO_3_/EMIM-TFSI[20] | Transistor | √ | × | 165 | N. A. | 9.6 |
| BP/Po_x_[21] | Transistor | √ | × | 122 | 78 | 2000 |
| MoS_2_/HfO_x_/ITO[8] | Transistor | √ | × | 117 | 35 | N. A. |
| Gr/AlO_x_[10] | Transistor Memristor | √ | × | 115 | 17.5 | N. A. |
| Gr/AlO_x_[9] | Transistor | √ | × | 113 | 14 | N. A. |
| PEDOT:PSS/KCl[22] | Transistor | √ | × | N. A. | N. A. | N. A. |
| Au/Ti/h-BN/Cu  Au/Ti/h-BN/Au[23] | Memristor | √ | × | N. A. | N. A. | 600 |
| Ag/ZHO:GOQDs/Ag[24] | Memristor | √ | × | 700 | 30 | 13.5 |
| Gr/LiClO_4_/PEO[25] | Memristor | √ | × | N. A. | 700 | 0.5 |
| Gr/2D Perovskite/Au[26] | Memristor | √ | × | N. A. | 20 | 0.4 |
| MoS_2_[27] | Memtransistor | √ | × | N. A. | 70 | N. A. |
| MoS_2_/PTCDA[28] | Transistor | √ | √ | 500 | 6000 | 10 |

**References**

[1] Alles, H., J. Aarik, J. Kozlova, et al., "Atomic layer deposition of high-k oxides on graphene"*.* *arXiv preprint arXiv:1109.4026,* 2011.

[2] Zhang, H., G. Arutchelvan, J. Meersschaut, et al., "MoS2 Functionalization with a Sub-nm Thin SiO2 Layer for Atomic Layer Deposition of High-κ Dielectrics"*.* *Chemistry of Materials,* 2017. **29**(16): p. 6772-6780.

[3] Zhao, C., C.Z. Zhao, M. Werner, et al., "Dielectric relaxation of high-k oxides"*.* *Nanoscale research letters,* 2013. **8**(1): p. 456.

[4] Lee, G.-H., X. Cui, Y.D. Kim, et al., "Highly stable, dual-gated MoS2 transistors encapsulated by hexagonal boron nitride with gate-controllable contact, resistance, and threshold voltage"*.* *ACS nano,* 2015. **9**(7): p. 7019-7026.

[5] Petrone, N., T. Chari, I. Meric, et al., "Flexible graphene field-effect transistors encapsulated in hexagonal boron nitride"*.* *ACS nano,* 2015. **9**(9): p. 8953-8959.

[6] Liu, Y., H. Wu, H.C. Cheng, et al., "Towards barrier free contact to MoS2 using graphene electrodes"*.* *arXiv preprint arXiv:1412.7718,* 2014.

[7] Wang, L., Z. Chen, C.R. Dean, et al., "Negligible environmental sensitivity of graphene in a hexagonal boron nitride/graphene/h-BN sandwich structure"*.* *ACS nano,* 2012. **6**(10): p. 9314-9319.

[8] Wang, X., H. Tian, S. Shen, et al., "MoS2 Synaptic Transistor With Tunable Weight Profile"*.* *IEEE Transactions on Electron Devices,* 2018. **65**(8): p. 3543-3547.

[9] Tian, H., W. Mi, X.-F. Wang, et al., "Graphene dynamic synapse with modulatable plasticity"*.* *Nano letters,* 2015. **15**(12): p. 8013-8019.

[10] Tian, H., W. Mi, H. Zhao, et al., "A novel artificial synapse with dual modes using bilayer graphene as the bottom electrode"*.* *Nanoscale,* 2017. **9**(27): p. 9275-9283.

[11] Yang, Y., Y. He, S. Nie, et al., "Light Stimulated IGZO-Based Electric-Double-Layer Transistors For Photoelectric Neuromorphic Devices"*.* *IEEE Electron Device Letters,* 2018. **39**(6): p. 897-900.

[12] van de Burgt, Y., E. Lubberman, E.J. Fuller, et al., "A non-volatile organic electrochemical device as a low-voltage artificial synapse for neuromorphic computing"*.* *Nature materials,* 2017. **16**(4): p. 414.

[13] Jiang, J., J. Guo, X. Wan, et al., "2D MoS2 Neuromorphic Devices for Brain‐Like Computational Systems"*.* *Small,* 2017. **13**(29): p. 1700933.

[14] Qin, S., F. Wang, Y. Liu, et al., "A light-stimulated synaptic device based on graphene hybrid phototransistor"*.* *2D Materials,* 2017. **4**(3): p. 035022.

[15] He, H.K., R. Yang, W. Zhou, et al., "Photonic Potentiation and Electric Habituation in Ultrathin Memristive Synapses Based on Monolayer MoS2"*.* *Small,* 2018. **14**(15): p. 1800079.

[16] Zhu, J., Y. Yang, R. Jia, et al., "Ion gated synaptic transistors based on 2D van der Waals crystals with tunable diffusive dynamics"*.* *Advanced Materials,* 2018. **30**(21): p. 1800195.

[17] Wang, Y., Z. Lv, J. Chen, et al., "Photonic Synapses Based on Inorganic Perovskite Quantum Dots for Neuromorphic Computing"*.* *Advanced Materials,* 2018: p. 1802883.

[18] John, R.A., F. Liu, N.A. Chien, et al., "Synergistic Gating of Electro‐Iono‐Photoactive 2D Chalcogenide Neuristors: Coexistence of Hebbian and Homeostatic Synaptic Metaplasticity"*.* *Advanced Materials,* 2018. **30**(25): p. 1800220.

[19] Sun, L., Y. Zhang, G. Hwang, et al., "Synaptic Computation Enabled by Joule Heating of Single-Layered Semiconductors for Sound Localization"*.* *Nano letters,* 2018. **18**(5): p. 3229-3234.

[20] Yang, C.S., D.S. Shang, N. Liu, et al., "A Synaptic Transistor based on Quasi‐2D Molybdenum Oxide"*.* *Advanced Materials,* 2017. **29**(27): p. 1700906.

[21] Tian, H., Q. Guo, Y. Xie, et al., "Anisotropic black phosphorus synaptic device for neuromorphic applications"*.* *Advanced Materials,* 2016. **28**(25): p. 4991-4997.

[22] Gkoupidenis, P., N. Schaefer, B. Garlan, et al., "Neuromorphic functions in PEDOT: PSS organic electrochemical transistors"*.* *Advanced Materials,* 2015. **27**(44): p. 7176-7180.

[23] Shi, Y., X. Liang, B. Yuan, et al., "Electronic synapses made of layered two-dimensional materials"*.* *Nature Electronics,* 2018. **1**(8): p. 458.

[24] Yan, X., L. Zhang, H. Chen, et al., "Graphene Oxide Quantum Dots Based Memristors with Progressive Conduction Tuning for Artificial Synaptic Learning"*.* *Advanced Functional Materials,* 2018: p. 1803728.

[25] Sharbati, M.T., Y. Du, J. Torres, et al., "Low‐Power, Electrochemically Tunable Graphene Synapses for Neuromorphic Computing"*.* *Advanced Materials,* 2018. **30**(36): p. 1802353.

[26] Tian, H., L. Zhao, X. Wang, et al., "Extremely Low Operating Current Resistive Memory Based on Exfoliated 2D Perovskite Single Crystals for Neuromorphic Computing"*.* *ACS nano,* 2017. **11**(12): p. 12247-12256.

[27] Sangwan, V.K., H.-S. Lee, H. Bergeron, et al., "Multi-terminal memtransistors from polycrystalline monolayer molybdenum disulfide"*.* *Nature,* 2018. **554**(7693): p. 500.

[28] Wang, S., C. Chen, Z. Yu, et al., "A MoS2/PTCDA Hybrid Heterojunction Synapse with Efficient Photoelectric Dual Modulation and Versatility"*.* *Advanced Materials,* 2019. **31**(3): p. 1806227.
